# The first complete mitochondrial genome of *Antigona lamellaris* (Schumacher, 1817) (Veneroida: Veneridae)

**DOI:** 10.1080/23802359.2021.1902406

**Published:** 2021-03-24

**Authors:** Shengping Zhong, Yan Jiang, Yonghong Liu, Guoqiang Huang, Xiuli Chen

**Affiliations:** a Guangxi Engineering Technology Research Center for Marine Aquaculture, Guangxi Institute of Oceanology Co., Ltd, Beihai, China; b Institute of Marine Drugs, Guangxi University of Chinese Medicine, Nanning, China; c Guangxi Key Laboratory of Aquatic Genetic Breeding and Healthy Aquaculture, Guangxi Academy of Fishery Sciences, Nanning, China

**Keywords:** Mitochondrial genome, *Antigona lamellaris*, Veneridae

## Abstract

Venus clams (Veneridae) including *Antigona lamellaris* are commercially important fishery resources by their dominance in local benthic communities. However, despite their great diversity, the phylogenetic and taxonomic relationships in venus clams remain poorly understood. In this study, we report the first complete mitochondrial genome of *A. lamellaris*. The mitogenome has 17,532 base pairs (67.9% A + T content) and is made up of a total of 37 genes (13 protein-coding, 22 transfer RNAs and 2 ribosomal RNAs), plus a putative control region. This study will provide useful molecular resources for clarifying taxonomic and phylogenetic confusion in venus clams.

The Bivalvia Veneridae, known as venus clams, is the most speciose diverse bivalve mollusks family containing more than 800 extant species around the world which were grouped into about 14 subfamilies (Kappner and Bieler 2006), many of which are ecologically and economically important due to their huge abundance in local benthic ecosystem (Chen et al. 2011), such as *Antigona lamellaris* (Schumacher 1817). *Antigona lamellaris* is an economically important fishery resource for its valuable nutrition as delicious seafood in Southeast China. Despite their high ecological and economic value, Veneridae have a confusing taxonomic history and their interrelationships become challenged using only morphology-based phylogenetic analyses due to their great diversity and morphological similarity(Mikkelsen et al. 2006). Complete mitochondrial genomes have been shown to powerful molecular tool in resolving phylogenetic relationship. However, up to now, the mitogenomic sequences of venus clams is still lack. Here, we report the first complete mitochondrial genome of venus clams *A. lamellaris*, which will provide a useful genetic resource for phylogenetic and evolutionary analyses in Veneridae.

Tissue samples of *A. lamellaris* from six individuals were collected from GuangXi province, China (Beihai, 21.543327 N, 109.583014 E) using local trawl nets, together with the whole body specimen (#GR0263) were deposited at Marine biological Herbarium, Guangxi Institute of Oceanology, Beihai, China (Dr. Zhong, shpzhong@foxmail.com). The total genomic DNA was extracted from the muscle of the specimens using an SQ Tissue DNA Kit (OMEGA, Guangzhou, China) following the manufacturer’s protocol. DNA libraries (350 bp insert) were constructed with the TruSeq NanoTM kit (Illumina, San Diego, CA) and were sequenced (2 × 150 bp paired-end) using HiSeq platform at BGI Company, China. Mitogenome assembly was performed by MITObim (Hahn et al. 2013). The cytochrome oxidase subunit I (COI) gene of *A. lamellaris* (GenBank accession number: DQ458472) was chosen as the initial reference sequence for MITObim assembly. Gene annotation was performed by MITOS (http://mitos2.bioinf.uni-leipzig.de).

The complete mitogenome of *A. lamellaris* (GenBank accession number: MT254059) is small in size (17,532 bp) within venus clams (Xu et al., 2012), which contains conserved set of 13 protein-coding genes (PCGs), 2 ribosomal RNA genes, 22 transfer RNA genes, and a putative control region. A total of 37 genes were annotated and 411 nucleotides were putative control region. The overall base composition of the mitogenome is estimated to be A 26.8%, T 41.2%, C 9.3% and G 22.7%, with a high A + T content of 67.9%, which is similar, but slight lower than *Dosinia altior* (69.57%) (Lv et al. 2018) which was closely related to *Antigona*. The phylogenetic analysis inferred from the concatenated nucleotides sequences of 13 PCGs also suggests that *Antigona* has closely relationship with *Dosinia* (Figure 1), which is consistent with the phylogenetic analyses of venus clams using nuclear and mitochondrial gene sequences (Kappner and Bieler 2006). The complete mitochondrial genome sequence of *A. lamellaris* was the first sequenced mitogenome in *Antigona*, which will be useful for better understanding the phylogenetic and taxonomic classification in venus clams.

**Figure 1.**
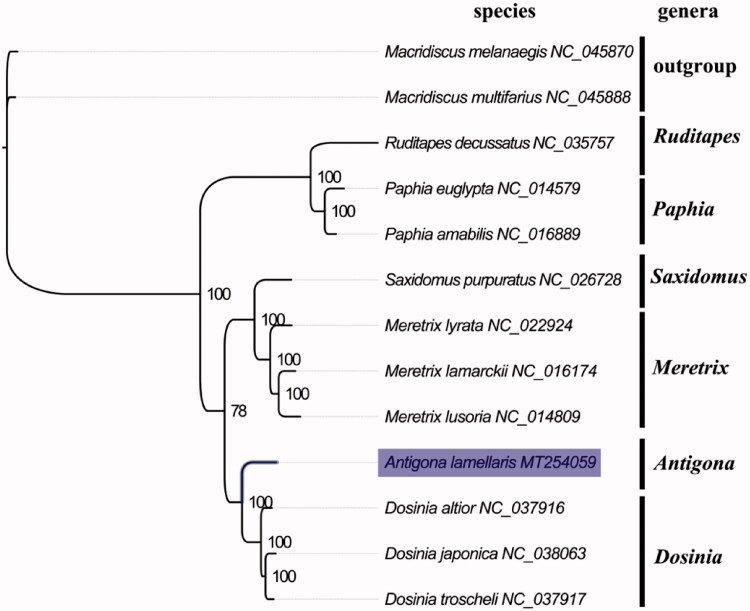
Phylogenetic tree of 13 species in family Veneridae. The complete mitogenomes were downloaded from GenBank and the phylogenic tree based on the concatenated nucleotide sequences of 13 mitochondrial PCGs was constructed by maximum-likelihood method with 100 bootstrap replicates. The bootstrap values were labeled at each branch nodes, Macridiscus melanaegis and Macridiscus multifarius were chosen to be outgroup species.

## Data Availability

The genome sequence data that support the findings of this study are openly available in GenBank of NCBI at (https://www.ncbi.nlm.nih.gov/) under the accession no. MT254059. The associated BioProject, SRA, and Bio-Sample numbers are PRJNA701318, SRR13684237, and SAMN17862031, respectively.
